# Design aspects of Bi_2_Sr_2_CaCu_2_O_8+δ_ THz sources: optimization of thermal and radiative properties

**DOI:** 10.3762/bjnano.12.103

**Published:** 2021-12-21

**Authors:** Mikhail M Krasnov, Natalia D Novikova, Roger Cattaneo, Alexey A Kalenyuk, Vladimir M Krasnov

**Affiliations:** 1 Keldysh Institute of Applied Mathematics of RAS, Moscow, Russia; 2 Moscow Institute of Physics and Technology, 141700 Dolgoprudny, Russia; 3 Department of Physics, Stockholm University, AlbaNova University Center, SE-10691 Stockholm, Sweden; 4 Institute of Metal Physics of National Academy of Sciences of Ukraine, 03142 Kyiv, Ukraine; 5 Kyiv Academic University, 03142 Kyiv, Ukraine

**Keywords:** high-temperature superconductivity, Josephson junctions, numerical modelling, terahertz sources

## Abstract

Impedance matching and heat management are important factors influencing the performance of terahertz sources. In this work we analyze thermal and radiative properties of such devices based on mesa structures of a layered high-temperature superconductor Bi_2_Sr_2_CaCu_2_O_8+δ_. Two types of devices are considered containing either a conventional large single crystal or a whisker. We perform numerical simulations for various geometrical configurations and parameters and make a comparison with experimental data for the two types of devices. It is demonstrated that the structure and the geometry of both the superconductor and the electrodes play important roles. In crystal-based devices an overlap between the crystal and the electrode leads to appearance of a large parasitic capacitance, which shunts terahertz emission and prevents impedance matching with open space. The overlap is avoided in whisker-based devices. Furthermore, the whisker and the electrodes form a turnstile (crossed-dipole) antenna facilitating good impedance matching. This leads to more than an order of magnitude enhancement of the radiation power efficiency in whisker-based, compared to crystal-based, devices. These results are in good agreement with presented experimental data.

## Introduction

Tunable, monochromatic, continuous-wave (CW), compact, and power-efficient terahertz (THz) sources of electromagnetic waves (EMW) are required for a broad variety of applications [1]. However, the key obstacle, colloquially known as “the THz gap” [1], is caused by a low radiation power efficiency (RPE) of THz sources. Despite a remarkable progress achieved by semiconducting quantum cascade lasers (QCL’s) [2–3], their RPE drops well below the percent level at low THz frequencies [4–6]. Furthermore, operation of QCLs is limited by the thermal smearing of quantum levels, which becomes significant at frequencies *f* 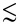 *k*_B_*T*/*h*, where *k*_B_ and *h* are the Boltzmann and the Planck constant, respectively, and *T* is the operation temperature. For room temperature, *T* = 300 K, this happens at *f* ≃ 6.25 THz. QCLs have to be cooled down in order to operate at significantly lower primary frequencies [4–6]. Although room temperature operation of QCLs at low frequencies can be achieved via mixing and down conversion of higher primary frequencies, this comes at the expense of dramatic reduction of RPE [2–3,5,7–8].

The layered high-temperature superconductor Bi-2212 (Bi_2_Sr_2_CaCu_2_O_8+δ_) may provide an alternative possibility for the creation of cryogenic CW THz sources [9–22]. Bi-2212 represents a natural stack of atomic scale intrinsic Josephson junctions (IJJs) [23–26]. Josephson junctions have an inherently tunable oscillation frequency, *f*_J_ = (2*e*/*h*)*V*, where *e* is electron charge and *V* is the bias voltage per junction. The frequency is limited only by the superconducting energy gap, which can be in excess of 30 THz for Bi-2212 [27–28]. A broad range tunability of emission in the whole THz range 1–11 THz has been demonstrated for small Bi-2212 mesa structures [14].

The operation of Josephson emitters is limited by two primary obstacles: self-heating and impedance mismatch. Josephson devices stop operating when their temperature exceeds the superconducting critical temperature *T*_c_. Self-heating in Bi-2212 mesa structures has been extensively studied [28–39]. Although *T*_c_ of Bi-2212 may be quite high, up to ≃95 K [28], self-heating is substantial due to the low heat conductance of superconductors. Self-heating limits the maximum bias voltage that can be reached without critical overheating of a mesa and, therefore, the maximum achievable frequency and emission power. Furthermore, as pointed out in [40], self-heating creates a general limitation for the maximal achievable emission power for any cryogenic device (not only superconducting). Taking into account the limited cooling power of compact cryorefrigerators (sub-watt at low *T*), a device with RPE ≈ 1% would not be able to emit significantly more than 1 mW. Therefore, larger emission power from cryogenic sources may only be achieved via enhancement of the RPE. The maximum achievable RPE is 50% in the case of perfect match between the device microwave impedance and that of open space [41]. However, the reported RPE of Bi-2212 THz sources is much smaller [14] due to a significant impedance mismatch. Therefore, improvement of THz sources requires a proper design of cooling elements, to handle self-heating, and impedance matching microwave antennas, to improve RPE.

In this work we analyze design aspects of THz sources based on Bi-2212 mesa structures. Thermal and radiative properties are studied for two types of devices containing either a conventional large single crystal or a needle-like whisker. We present numerical simulations for various geometrical configurations and parameters and make a comparison with experimental data. It is demonstrated that the structure and the geometry of both the superconductor and the electrodes play important roles. Electrodes provide an effective heat sink channel and help in the reduction of self-heating. They also influence radiative properties. However, this influence is opposite for crystal-based (worsening) and whisker-based (improvement) devices. The superconductor geometry is also crucial. Devices based on large crystals suffer from a large parasitic capacitance at the overlap between the crystal and the electrodes. It prevents good impedance matching and reduces RPE. The overlap is avoided in whisker-based devices. Moreover, the whisker itself, together with the electrodes, forms a turnstile (crossed-dipole) antenna, facilitating good impedance matching. We show that this can lead to more than an order of magnitude enhancement of RPE, compared to crystal-based devices. Those results are in good agreement with experimental data, which demonstrate that THz emission from whisker-based devices is much larger than from crystal-based devices with the same geometry.

## Experimental Results

**Figure 1:**
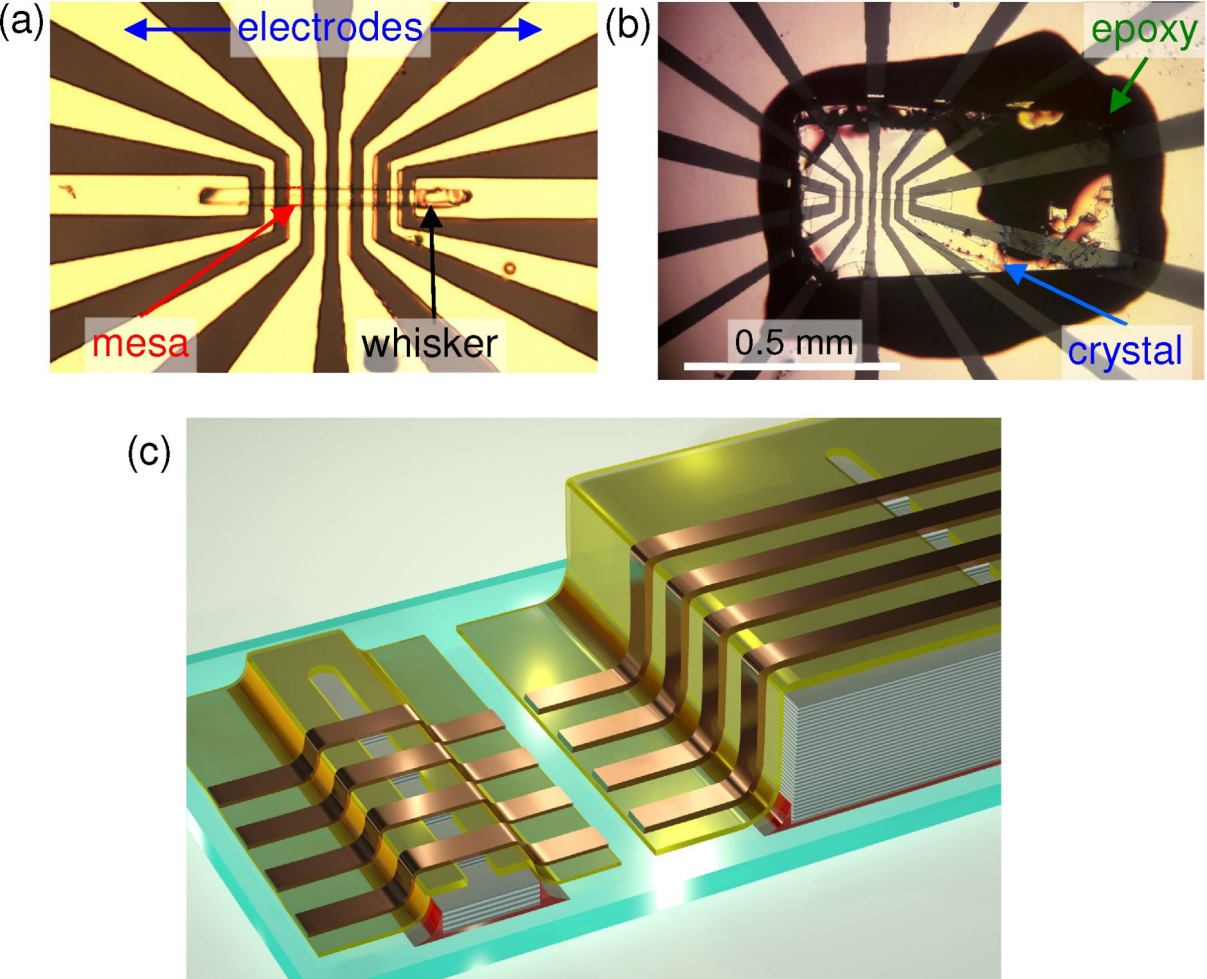
Optical images of (a) a whisker and (b) a crystal-based device with similar electrode geometries. (c) A sketch of both devices.

Figure 1a,b show optical images of two studied devices. They have a similar geometry and were fabricated using the same procedure. The main difference is that the device in panel (a) was made using a whisker while that in panel (b) was made using a conventional single crystal. Figure 1c shows sketches of both devices. Bi-2212 whiskers have typical aspect ratios of 100:10:1 in the *a*, *b*, and *c* crystallographic directions, respectively [42]. Our whiskers have typical dimensions of several hundreds of micrometers along *a*, 20–40 μm along *b*, and just a few micrometers along *c*. The conventional single crystal is much larger with sizes of almost a square millimeter in the *ab*-plane and several hundreds of micrometers in the *c*-direction.

The fabrication process starts by gluing a corresponding crystal on a 5 × 5 mm^2^ sapphire substrate using epoxy glue. The crystal is cleaved under ambient conditions. After that the sample is immediately put into a deposition chamber and a protective gold layer of 60–80 nm is deposited to avoid surface passivation. Next, a line pattern of photoresist is written with a length of 100–200 μm and a width of 5–15 μm on a flat portion of Bi-2212 surface, followed by argon-ion etching of the unprotected parts of Au and Bi-2212, the deposition of insulating SiO_2_ or CaF_2_ layers and a lift-off of the photoresist at the line. The depth of Bi-2212 etching at this stage (*d*_m_ ≈ 200–400 nm) defines the height of mesas and the number of IJJ in the device, *N* = *d*_m_/*s*, where *s* ≃ 1.5 nm is the interlayer spacing between double CuO_2_ layers in Bi-2212. After that a top Ti/Au layer with a total thickness of approx. 200 nm is deposited. Finally, several electrodes, crossing the line in a perpendicular direction, are made by photolithography and argon-ion etching. Mesa structures are formed at the overlap between the line and the electrodes, as indicated in Figure 1a.

**Figure 2:**
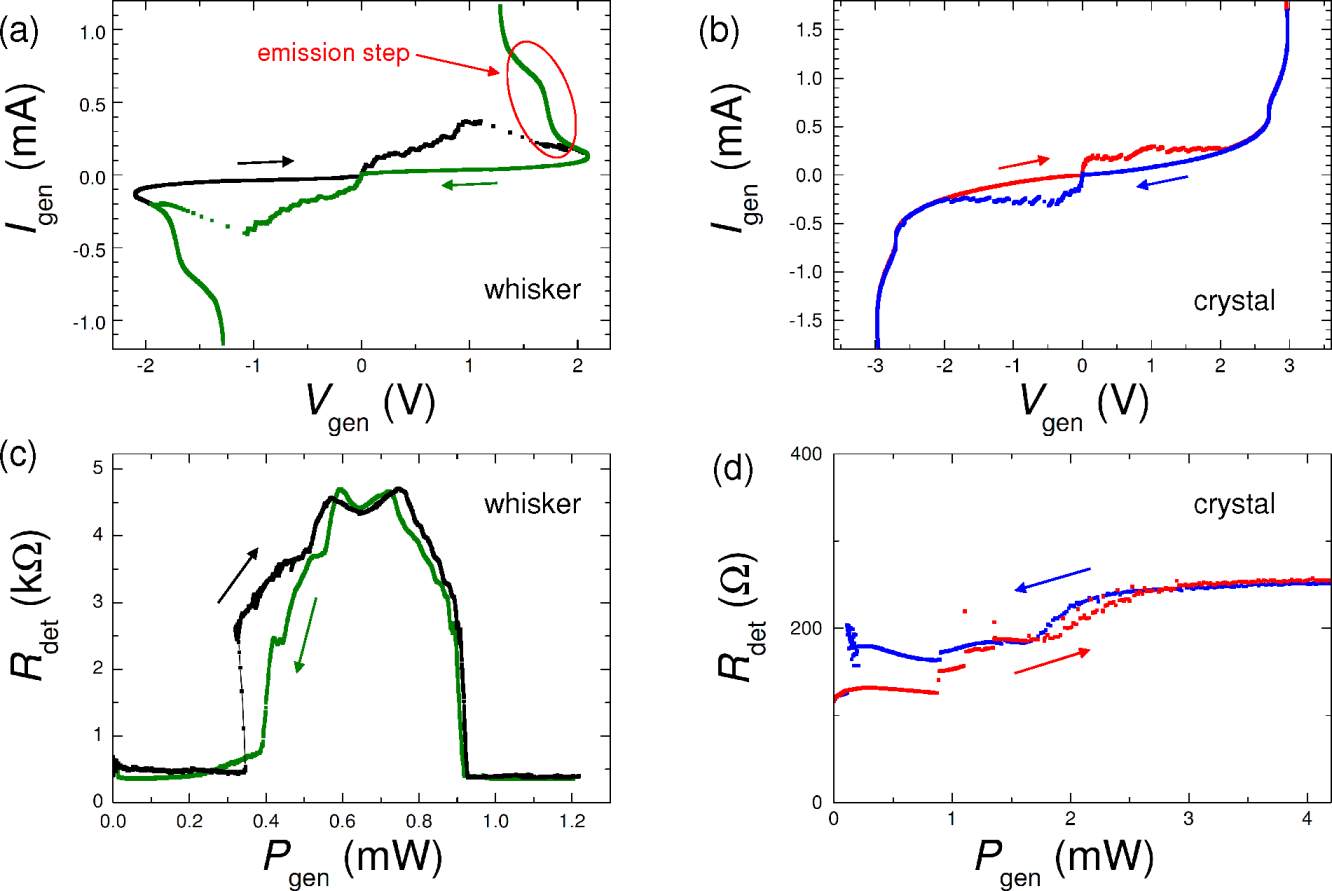
Current–voltage characteristics of mesa structures on (a) whisker- and (b) crystal-based devices. (c, d) On-chip generation–detection experiment for (c) whisker- and (d) crystal-based devices. Here, the ac resistance of the detector mesa is shown as function of the total dc dissipation power, *P*_gen_, of the generator mesa. For the whisker-based device (c) a profound emission occurs at the step in the *I*–*V* marked in panel (a). For the crystal-based device (d) only a small monotonic increment of *R*_det_ vs *P*_gen_ is observed, caused by gradual self-heating.

Figure 2a,b shows current–voltage (*I*–*V*) characteristics of mesas of whisker- and crystal-based devices, respectively. The *I*–*V* curves are fairly similar. They contain multiple branches due to one-by-one switching of IJJs from the superconducting to the resistive state. The are *N* ≈ 200 and ≈300 IJJs in whisker and crystal mesas, respectively. Both the whisker and the crystal have a similar suppressed *T*_c_ ≈ 65–70 K and low critical current densities of IJJs, *J*_c_ ≈ 100 A/cm^2^, indicating a strongly underdoped state of Bi-2212 [43].

Radiative properties of our whisker-based devices were analyzed in [40]. A significant EMW emission at *f* ≃ 4.2 THz with a record-high RPE reaching 12% was reported. The emission occurs at the step in the *I*–*V* marked in Figure 2a. In Figure 2c we show results of in situ THz generation-detection experiment on a whisker-based device. We follow the procedure developed in [14], where details of the technique can be found. We use one mesa with the *I*–*V* like in Figure 2a as a generator, and another mesa on the same device as a switching current detector. The detector mesa is biased by a small alternating current and the generator by a direct current in the same range as in Figure 2a. Figure 2c shows the ac resistance of the detector mesa, *R*_det_, as a function of dissipation power in the generator mesa, *P*_gen_ = *I*_gen_*V*_gen_. It is anticipated that self-heating is monotonic (approximately linear) with the dissipation power, while the emission is nonmonotonic [14,40] because it occurs at certain bias voltages, corresponding to geometrical resonances in the mesa [14,16,24,26]. From Figure 2c it is seen that a profound EMW emission occurs in a whisker-based mesa, leading to more than an order of magnitude enhancement of *R*_det_. The emission occurs in a specific bias range, corresponding to the step in the *I*–*V* marked in Figure 2a. To avoid repetitions we address the reader to [40] for more details.

In Figure 2d we show similar generation–detection data for the crystal-based device from Figure 1b and Figure 2b. In contrast to the whisker-based device, here we observe only a small monotonic increment of *R*_det_ with increasing *P*_get_, which is the consequence of self-heating. On top of it there may be a small non-monotonic signal at 0.5 mW 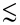 *P*_gen_ 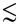 1.5 mW, which can be attributed to THz emission. This is qualitatively similar to results reported earlier for small mesas on crystal-based devices [14]. For whisker-based mesas the ratio between emission and self-heating responses is quantitatively different: The emission peak *R*_det_(*P*_get_) is much larger than the monotonic self-heating background. This indicates a much larger RPE in whisker-based devices [40].

## Numerical Results

To understand the reported difference between crystal- and whisker-based devices and to suggest possible optimizations of THz sources, we performed numerical modelling using the 3D finite element software Comsol Multiphysics. Below, we present simulations of thermal and radiative properties calculated using “Heat Transfer” and “RF” modules, respectively. The presented simulations contain several simplifications and, therefore, are not aiming to self-consistently predict the extent of self-heating, Δ*T*, or the radiative power. Their goal is to reveal general trends and geometrical factors contributing to design aspects of Bi-2212 THz sources.

### Modelling of heat transfer

Accurate analysis of self-heating in Bi-2212 mesas is a complex non-linear problem [28,30–32,36,38]. Simulations presented below are made for the base temperature *T*_0_ = 10 K and for sizes similar to the actual devices, shown in Figure 1: substrate 5 × 5 × 0.3 mm^3^, crystal 1 × 1 × 0.3 mm^3^, whisker 300 × 30 × 3 μm^3^ and mesa 30 × 30 × 0.3 μm^3^. The thickness of gold electrode is 200 nm. The thickness of epoxy layer, *d*_e_, depends on the quantity of applied glue, area of the crystal, experience, and luck. For whisker devices we managed to reduce it to *d*_e_ 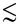 1 μm. To do so we use a tiny amount of epoxy and also squeeze it out by pressing the whisker upon gluing. This procedure is effective for whiskers due to their small area. For large crystals, requiring more epoxy, this is more difficult and the remaining epoxy layer is usually thicker. For this reason we assume the epoxy thickness *d*_e_ = 1 μm for whisker and *d*_e_ = 5 μm for crystal-based devices.

The monocrystalline sapphire substrate has a very good thermal conductivity, κ, at low *T*. The substrate is thermally well anchored with the boundary condition at the bottom surface *T* = *T*_0_. Due to the large κ, temperature variation in the substrate is negligible. Therefore, we use a constant κ = 3000 W·K^−1^·m^−1^ for the substrate, corresponding to a monocrystalline sapphire at *T* ≃ 10 K [44]. In contrast, the epoxy used for gluing Bi-2212 crystals has a poor heat conductance at low *T*. We do not consider its *T*-dependence because it acts just as a heat blocking layer, which we assume to have κ_e_ = 0.0025 W·K^−1^·m^−1^. However, it is necessary to take into account actual κ(*T*) dependencies of the other two materials, namely Bi-2212 and polycrystalline gold electrodes. At low *T* both have linear κ(*T*). For Bi-2212 we assume κ*_ab_*(*T*) = 0.1 *T*(*K*) W·K^−1^·m^−1^ [45] with an anisotropy κ*_ab_*/κ*_c_* = 8 [46]. For a polycrystalline gold thin film we use κ(*T*) = 3 *T*(*K*) W·K^−1^·m^−1^ [32]. The heat is produced in the mesa volume with a total power of 1 mW and uniform density.

**Figure 3:**
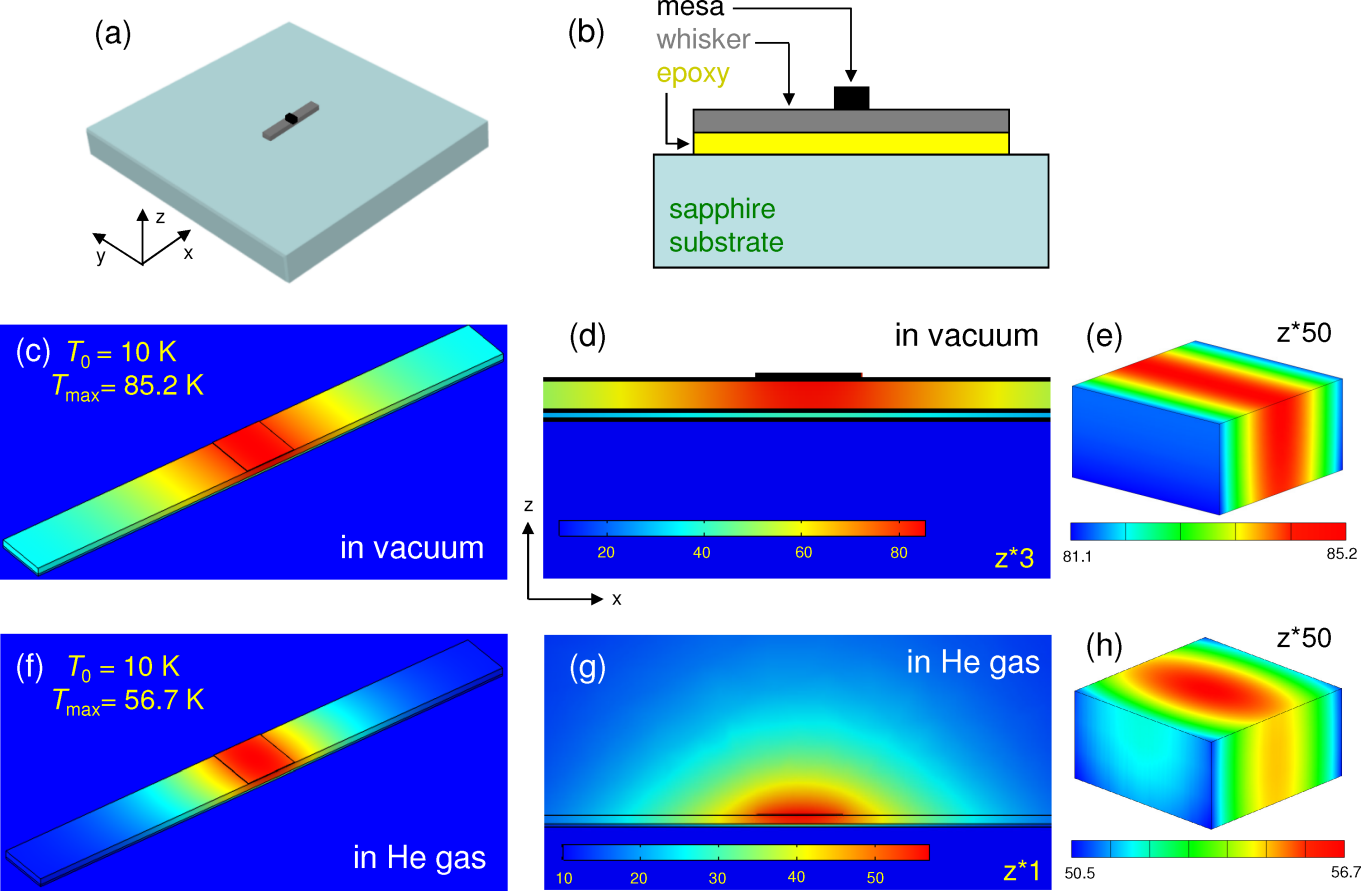
Heat transport in a whisker-based device without electrodes. (a) A sketch of the device and (b) a cross section through the mesa (not to scale). (c–e) Calculated temperature distribution for the device in vacuum. (f–h) The same for the device in exchange He gas.

Figure 3 represents heat transfer simulations for a whisker without an electrode. Figure 3a,b shows sketches of the device and the *x*–*z* cross-section through the mesa (not to scale), respectively. Figure 3c–e shows the temperature distribution for the case when the sample is placed in vacuum. Figure 3c shows the top view, Figure 3d the *x*–*z* cross section through the mesa (stretched by a factor of three in the vertical direction), and Figure 3e shows the temperature distribution in the mesa (stretched by a factor of 50 in the vertical direction). In this case the heat can only sink into the substrate. As seen from Figure 3d, the epoxy layer between the substrate and the whisker blocks heat flow into the substrate and causes substantial heating of the whole whisker with the maximum temperature in the center of the mesa reaching *T*_max_ = 85.2 K. Figure 3f–h show simulations for the same device in exchange ^4^He gas. Clearly, it helps to cool down the device, although self-heating still remains substantial, *T*_max_ = 56.7 K.

**Figure 4:**
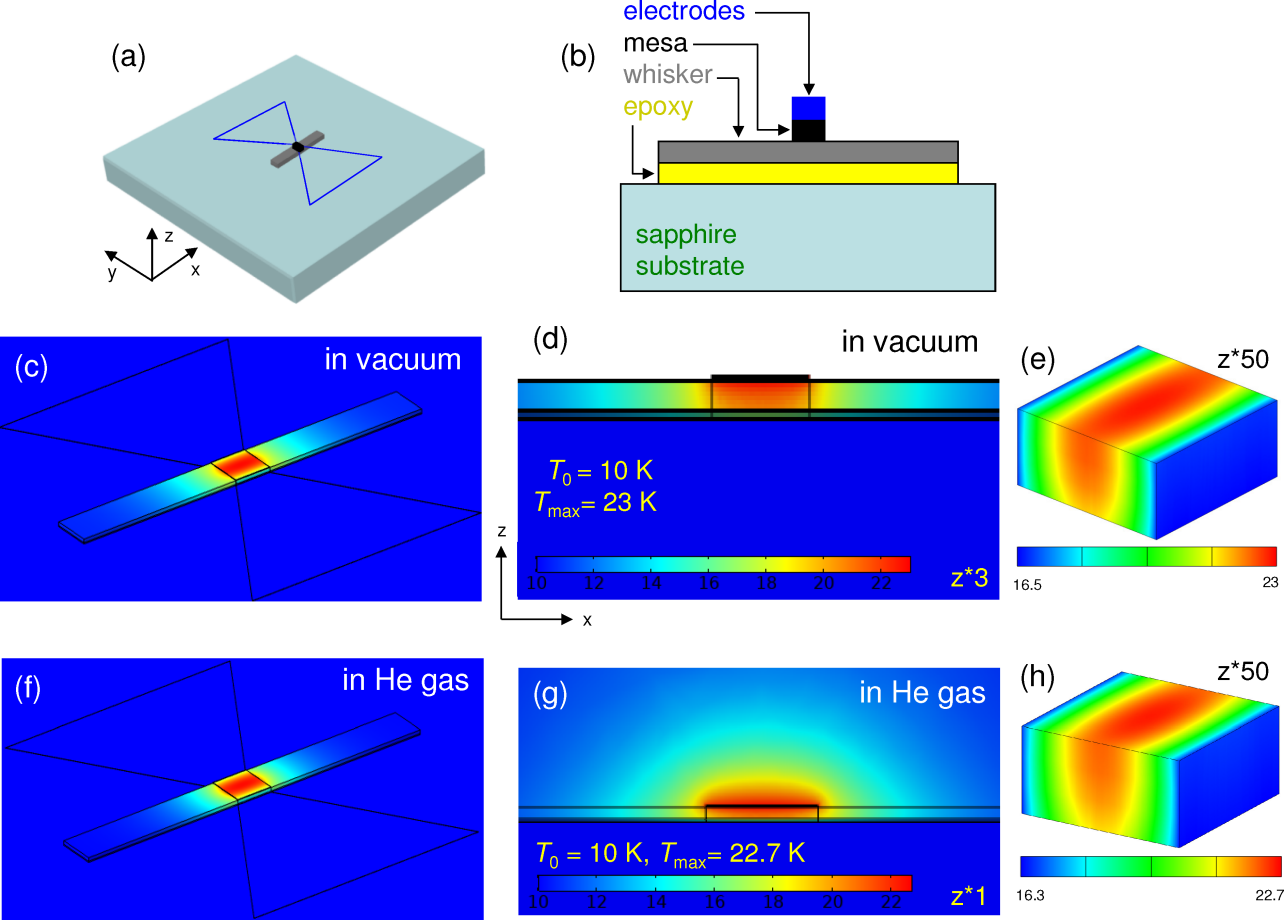
Heat transport in a whisker-based device with an electrode. (a) A sketch of the device and (b) a cross section through the mesa (not to scale). (c–e) Calculated temperature distribution for the device in vacuum. (f–h) The same for the device in exchange He gas.

Figure 4 shows simulations for the whisker-based device with a top Au electrode. Outside the whisker the electrode is in a direct contact with the sapphire substrate (no epoxy). This creates a good thermal sink and, as a result, *T*_max_ falls to ≈23 K. Addition of an exchange gas does not play a major role in this case because the main heat sink channel is provided by the electrode [33–34] acting as a heat spreading layer [29].

**Figure 5:**
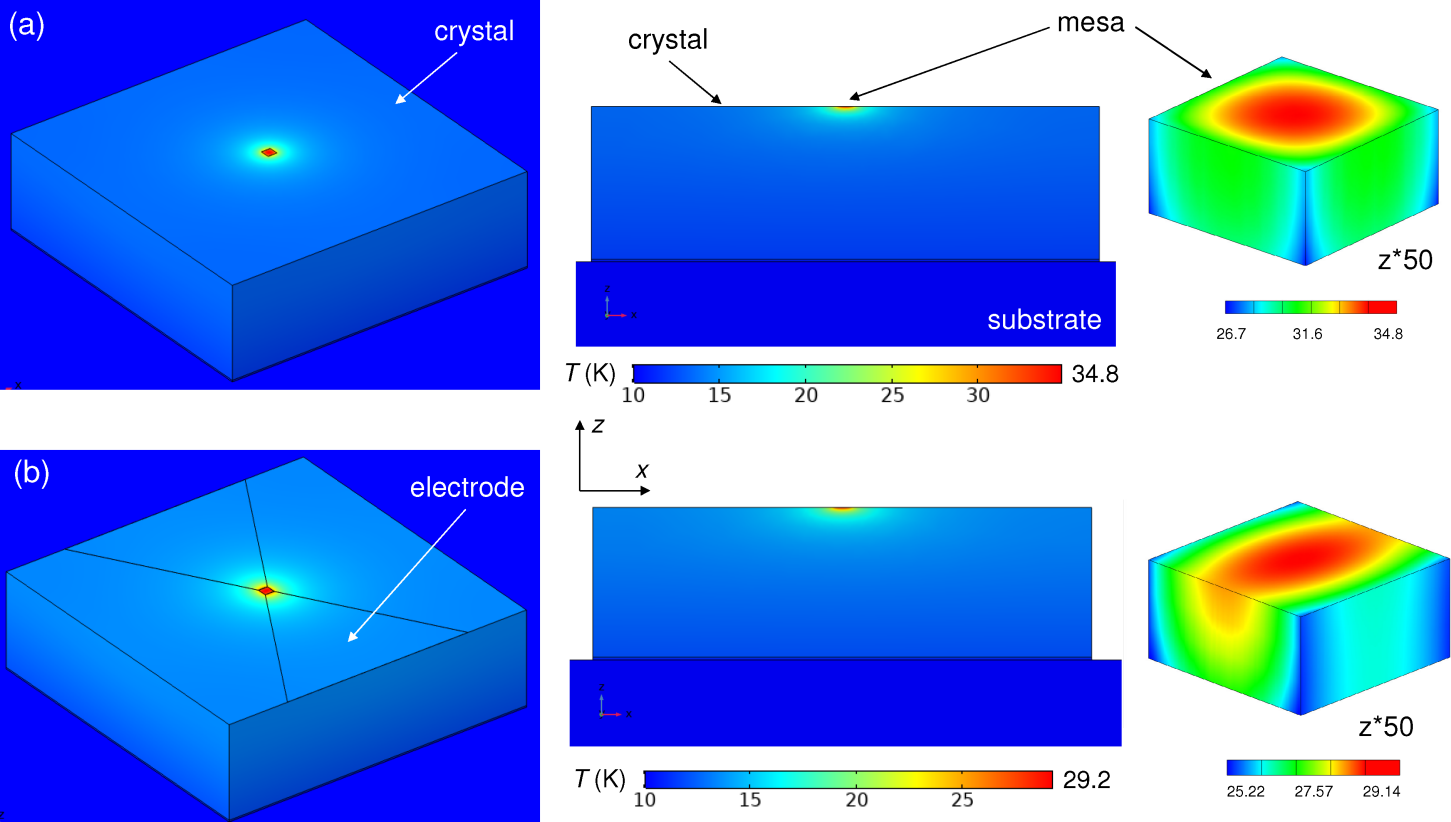
Heat transport in a crystal-based device in vacuum (a) without electrodes, (b) with electrodes. The left panels represent top views, the middle panels the *x*–*z* cross section through the mesa, and the right panels the mesa (expanded by factor of 50 in the *z*-direction).

Figure 5 shows the temperature distribution in a crystal-based device in vacuum without electrodes (Figure 5a) and with electrodes (Figure 5b). The main difference is that unlike in the whisker-device, Figure 3, there is no major temperature jump in the epoxy layer between the crystal and the substrate. This occurs because the heat resistance, *R*_h_ = *d*/(κ*A*), is inversely proportional to the area *A*. Due to a much larger crystal area, *R*_h_ of epoxy is negligible despite a low κ and larger *d*_e_ = 5 μm. Adding an electrode and He exchange gas further reduces self-heating, but their effect is not as profound as for the whisker-device, Figure 4, due to the effective heat sink channel into the substrate.

In many cases, self-heating is dominated by some bottlenecks. The origin of blocks to the heat flow is clearly revealed from inspection of the thermal gradients in the mesa where the heat is produced and by estimation of heat resistances of different elements. For example, from Figure 3e it is seen that for a bare mesa on a whisker the heat is flowing along the whisker. This occurs because the epoxy layer with a large *R*_h_ = 44.4 K/mW blocks direct (vertical) heat flow into the substrate. However, the maximal self-heating, Δ*T* = 75.2 K, is almost two times larger, implying that there is yet another bottleneck. It is caused by a small *bc* (*yz*) cross sectional area of the whisker. This additional in-plane heat resistance, *R*_h_ ≈ 30 K/mW, corresponds to the effective length of heat spreading along the whisker comparable to the size of the mesa, as can be seen from Figure 3d. For a whisker mesa with an electrode the thermal gradient changes the direction, see Figure 4e, indicating that the heat is flowing predominantly along the electrode. For comparison, the *c*-axis heat resistance of the mesa and the whisker are only 1.3 K/mW at *T* = 20 K. This implies that a significant reduction of self-heating in whisker devices could be achieved by replacing epoxy with a better heat-conducting material, for example, by soldering [10].

For a mesa on a crystal, Figure 5, the thermal gradient is fairly spherical (taking into account the anisotropy κ*_ab_*/κ*_c_* = 8). In this case self-heating is dominated by the spreading heat resistance in the crystal [29,31], *R*_h_ ≃ 
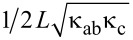
 = 23.6 K/mW at *T* = 20 K, where *L* = 30 μm is the in-plane size of the mesa. For comparison, the heat resistance of epoxy is only 2 K/mW for *d*_e_ = 5 μm. Consequently, epoxy is not the major problem for crystal devices (unless it is very thick *d*_e_ *>* 50 μm). For a real device self-heating will depend on the actual geometry, thicknesses, and material parameters. However, our analysis indicates that the optimization is much more important and efficient for whisker devices. This is caused by the low intrinsic *c*-axis heat resistance of whiskers due to the small thickness.

### Modelling of radiative properties

For the calculation of THz properties, a mesa (the source) is modelled as a lumped port with a fixed voltage amplitude. Unlike the heat transfer problem, this problem is linear so that the results directly scale with the source amplitude. To ease the understanding, we use an amplitude of 1 V. Simulations are made in a sphere with the radius *R*, which is chosen to be at least two times larger than the largest device size and the wavelength in vacuum. A perfectly matching layer with the thickness 0.1*R* is added outside the sphere to avoid reflections. We checked that the presented results do not depend on *R* and, therefore, properly describe far-field characteristics.

**Figure 6:**
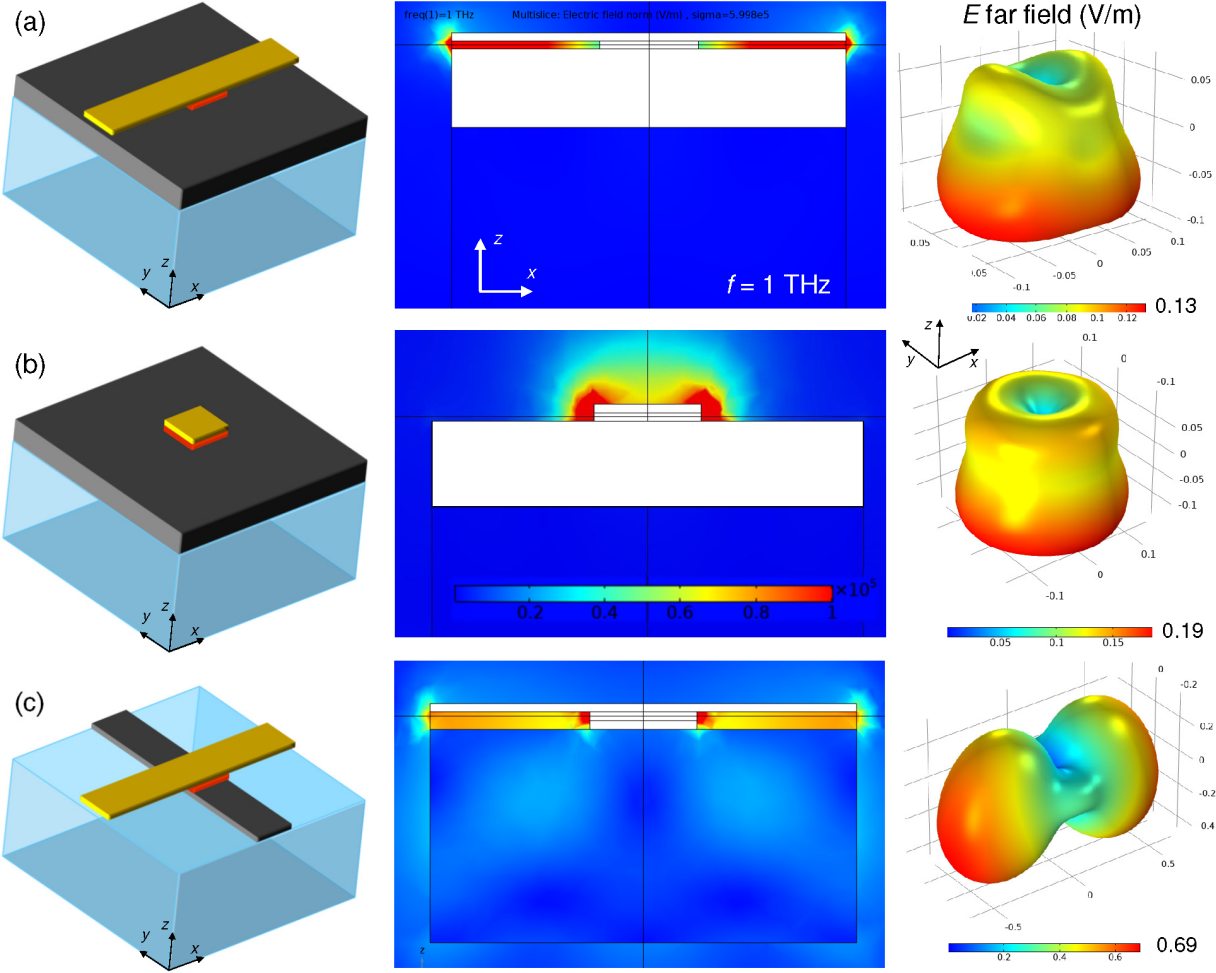
Simulated radiative properties at *f* = 1 THz for (a) crystal based device, (b) crystal-based device without electrodes, and (c) whisker-based device. Left panels show sketches of devices; middle panels – electric field amplitudes in the *x*-*z* cross-section through the mesa; right panels represent radiation patterns for the electric field amplitude in the far-field (outside the simulation sphere). Note a strong field concentration between the crystal and the electrode in (a).

Figure 6 shows radiative characteristics for three device geometries, sketched in the leftmost panels: Figure 6a shows a mesa (red) on a large crystal (black) with an attached metallic electrode (yellow), mounted on a dielectric substrate; Figure 6b shows a mesa on a large crystal with a capping metallic layer, without electrode; Figure 6c shows a mesa on a thin whisker (black) with an attached electrode. Simulations are performed for *f* = 1 THz and the sizes are selected relative to the wavelength in vacuum, λ_1_ = 300 μm: the substrate and the in-plane crystal size as well as whisker and electrode lengths are λ_1_/2 = 150 μm; the substrate height is λ_1_/4 = 75 μm; the in-plane mesa size, as well as whisker and electrode widths are λ_1_/8 = 37.5 μm; the crystal height is λ_1_/10 = 30 μm; mesa and whisker heights and the electrode thickness are λ_1_/100 = 3 μm; the simulation sphere radius is *R* = 2λ_1_ and the perfectly matching layer thickness is 0.2λ_1_. The sizes and parameters are chosen to be similar (but not identical) to studied samples in order to optimize the mesh size and the calculation time. Therefore, such simulations serve for a qualitative illustration of the difference between crystal- and whisker-based devices and the role of the electrodes. The conductivity of electrode and whisker is set to ≃6 × 10^5^ (Ω·m)^−1^ and the relative dielectric permittivity of the substrate is ε_r_ = 10. First we consider the case without dielectric losses, tan(δ) = 0. The middle panels in Figure 6 show the local distributions of electric field amplitudes in the *xz* crosssection through the mesa. The same color scale is used, indicated in the middle panel of Figure 6b. The rightmost panels represent far-field radiation patterns (directionality diagrams) of the electric field amplitude outside the simulation sphere.

From comparison of the middle panels in Figure 6a,b it can be seen that the electric field distribution is significantly different. In the crystal-based device the field is locked between the electrode and the crystal. This occurs because the electrode is laying on top of the crystal, forming together a parallel plate capacitor. The field is trapped inside this capacitor and goes neither in the substrate, nor into open space in the top hemisphere (with the exception of small edge fields). If we take a realistic specific capacitance of 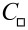 ≈ 0.1–1 fF/μm^2^ and an electrode area of 37.5 × 150 μm^2^, we obtain for *f* = 1 THz that the capacitive impedance is very small |*Z_C_*| = 1/2π*fC* ≃ 0.03–0.3 Ω, much smaller than the wave impedance of the free space, 
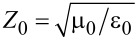
 ≃ 377 Ω. This leads to trapping of EMW in the electrode/crystal capacitance, which shunts open space and prevents emission.

To the contrary, for the whisker-based device, Figure 6c, the field goes out of the mesa as can be seen from the brighter overall tone of the pattern in the middle panel. The EMW propagation is particularly well seen in the bottom hemisphere due to formation of a standing wave pattern in the substrate. It is induced by reflections at the substrate/vacuum interfaces caused by a significant difference in refractive indices. Emission of EMW is associated with a cross-like structure of the whisker device, as sketched in the leftmost panel of Figure 6c. It obviates direct overlap of the whisker and the electrode and prevents the appearance of the large parasitic capacitance. This cross-like structure resembles the turnstile (crossed-dipole) antenna geometry, which facilitates good impedance matching with open space.

The difference between crystal- and whisker-based devices is also reflected in the far-field characteristics, shown in the rightmost panels of Figure 6a and Figure 6c. The maximum field amplitudes, *E*_max_, given in the bottom-right corners, are significantly different: 0.13 V/m for crystal- and 0.69 V/m for whisker-based device. Since the emitted power is proportional to 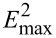, the RPE of the whisker-based device is almost 30 times larger than that of the crystal-based device. This indicates a good impedance matching for the whisker device and a poor matching for the crystal device. To further demonstrate the detrimental role of the parasitic electrode/crystal capacitor, in Figure 6b we considered the case with a mesa on a crystal without electrode and only with a capping top layer on the mesa. Such configuration is relevant for large mesas, contacted by a bonding wire [9]. Remarkably, the far-field emission is larger, *E*_max_ = 0.19 V/m, in the absence of the electrode. This clearly shows that the electrode on top of the crystal does not help in impedance matching. To the contrary, it makes things worse due to formation of the large parasitic capacitance shunting the EMW.

**Figure 7:**
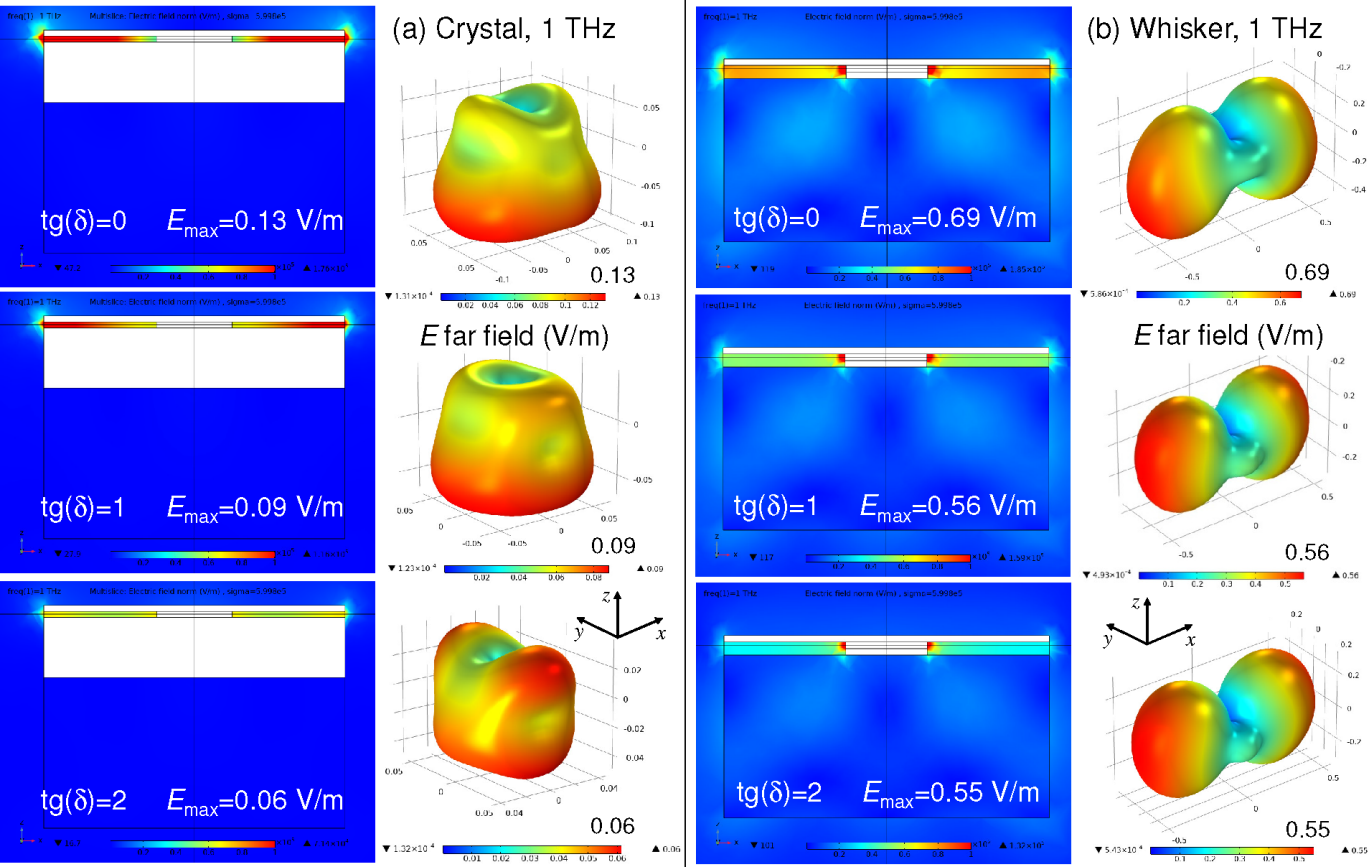
Variation of radiative properties with increasing dielectric losses tan(δ) = 0 (top row), 1 (middle row), and 2 (bottom row) for (a) crystal-based (two leftmost columns) and (b) whisker-based devices (two rightmost columns). Simulations are made at *f* = 1 THz. Note a rapid suppression of the far-field amplitudes in crystal-based devices.

Simulations presented in Figure 6 are made for ideal dielectrics with tan(δ) = 0. The detrimental role of the parasitic crystal/electrode capacitance becomes much more pronounced if we take into account dielectric losses, which can be significant at THz frequencies. In Figure 7 we show the variation of radiative properties of crystal-based (Figure 7a) and whisker-based devices (Figure 7b) upon increasing dielectric losses in the insulating layer between the crystal and the electrode for the crystal-based device and between substrate and electrode for the whisker-based device: tan(δ) = 0 (top), tan(δ) = 1 (middle), and tan(δ) = 2 (bottom row of panels). It is seen that for the whisker-based device dielectric losses only slightly reduce *E*_max_ from 0.69 V/m for tan(δ) = 0 to 0.55 V/m for tan(δ) = 2. For the crystal-based device the relative reduction is significantly larger, from 0.13 V/m for tan(δ) = 0 to 0.06 V/m for tan(δ) = 2. As a result, the ratio of RPE for whisker and crystal devices increases from ≈28 for tan(δ) = 0, to ≈39 for tan(δ) = 1 and ≈84 for tan(δ) = 2. This is a direct consequence of the electric field concentration in the parasitic crystal/electrode capacitance of crystal-based devices.

## Discussion

Josephson oscillators can provide unprecedented tunability in the whole THz range at a primary frequency [14]. However, being cryogenic devices, they are susceptible to self-heating, which limits both the achievable frequency range and the emission power. As pointed out in [40], the maximum emission power is limited by the cooling power of the device and the radiation power efficiency:


[1] $$P_{\text{THz}}<P_{\text{cooling}}\times \text{RPE }.$$

Enhancement of the effective cooling power requires implementation of special cooling elements in the device. Despite a significant progress in this direction [10,12–13,35–36,38,47], it is unlikely that a single emitter would be able to sustain the dissipation power above few tens of milliwatts. The tolerable dissipation power can be significantly enhanced by spreading it between several smaller emitters [10,19] because smaller mesa structures are less prone to self-heating [14,28–29,31]. Such a strategy has been successfully proved for arrays of Josephson junctions [48–50], for which coherent emission from up to 9000 synchronized junctions was reported [49]. Yet, the ultimate dissipation power is limited by the cooling power of the cryostat itself. For compact cryorefrigerators it is in the range of 100 mW. As follows from Equation 1, a source with RPE = 1% (which is good for THz sources) would not be able to emit more than *P*_THz_ = 1 mW. Therefore, further enhancement of the emission power requires enhancement of RPE. This, in turn, requires proper microwave design to facilitate impedance matching with open space. The maximum RPE in case of perfect matching is 50% [41], implying that up to 50 mW emitted THz power could be achieved.

Above we considered design aspects of THz sources, which contribute to obviation of self-heating and improvement of impedance matching. Several geometries of Bi-2212 devices were analyzed. It is shown that geometries of both the Bi-2212 crystal and the electrodes play important roles. Their effect, however, depends on the device type.

For crystal-based devices with large crystals of approx. 1 × 1 mm^2^ in the *ab*-plane, see Figure 1b, the size of the crystal plays contradicting roles in device operation. On the one hand, a large *ab*-plane area helps to spread heat into the substrate and reduces self-heating of the device, as seen from Figure 5. On the other hand, it leads to a large overlap area between the crystal and the top electrode. This creates a large parasitic capacitance that shunts THz emission and suppresses RPE.

In whisker-based devices the situation is different. Here the electrode provides the main heat sink channel, as shown in Figure 4. In general, our analysis indicated that self-heating optimization is much more important and efficient for whisker devices due to the low intrinsic *c*-axis heat resistance (caused by the small thickness of the whisker). Furthermore, the cross-like geometry prevents an overlap between the whisker and the electrode, thus obviating the parasitic capacitance. Moreover, the long whisker and the electrode act as two arms of a turnstile (crossed-dipole) antenna, facilitating good impedance matching with open space. Operation of whisker-based devices [22,40] and devices based on stand-alone mesas with similar cross-like electrodes [16] has been demonstrated by several groups.

The role of the substrate is also different. In crystal-based devices the large superconducting crystal screens the EMW, so that there is practically no field in the substrate, see Figure 6a,b. In this case, the substrate does not influence radiative properties. To the contrary, for a whisker-based device a significant fraction of EMW is going into the substrate due to its larger dielectric constant. The difference of dielectric constants of the substrate and vacuum leads to internal reflections and the formation of standing waves in the substrate, see Figure 6c. Therefore, the substrate acts as a dielectric resonator and may strongly affect the radiation pattern of the device.

The presented numerical simulations provide a qualitative explanation of the reported difference in radiative properties of whisker- and crystal-based devices, shown in Figure 1a,b. They explain why the RPE of whisker-based devices is much larger (by more than an order of magnitude, as follows from Figure 7). Those conclusions are in agreement with experimentally reported RPE values, which are in the range of 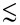
1% for crystal-based [10,14] and up to 12% for whisker-based [40] devices.

## Conclusion

To conclude, intrinsic Josephson junctions in the layered high-temperature superconductor Bi-2212 can provide an alternative technology for the creation of tunable, CW THz sources. In this work we analyzed two main phenomena that limit performance of such devices: self-heating and low RPE caused by impedance mismatch. We presented numerical simulations of thermal and radiative properties of Bi-2212 THz sources based on conventional large single crystals and needle-like whiskers. Simulations are performed for various geometrical configurations and parameters. A comparison with experimental data for crystal- and whisker-based devices is made. It is demonstrated that the structure and the geometry of both the superconductor and the electrodes play important roles. Crystal-based devices suffer from a large parasitic capacitance due to an overlap between the crystal and the electrodes. This prevents good impedance matching and reduces RPE. The overlap is avoided in whisker-based devices. Moreover, the whisker and the electrodes form a turnstile (crossed-dipole) antenna facilitating good impedance matching with open space. Our simulations demonstrate that this may enhance the radiation power efficiency in whisker-based devices by more than an order of magnitude compared to crystal-based devices, which is consistent with experimental data.
